# Genetic Markers of *Helicobacter pylori* Resistance to Clarithromycin and Levofloxacin in Moscow, Russia

**DOI:** 10.3390/cimb46070397

**Published:** 2024-06-29

**Authors:** Natalia Bodunova, Larisa Tsapkova, Vera Polyakova, Irina Baratova, Konstantin Rumyantsev, Natalia Dekhnich, Karina Nikolskaya, Margarita Chebotareva, Irina Voynovan, Elena Parfenchikova, Galina Pronina, Ekaterina Chernikova, Dmitry Bordin

**Affiliations:** 1 A.S. Loginov Moscow Clinical Scientific Center, 111123 Moscow, Russia; v.polyakova@mknc.ru (V.P.); i.baratova@mknc.ru (I.B.); k.rumyantsev@mknc.ru (K.R.); k.nikolskaya@mknc.ru (K.N.); m.chebotareva@mknc.ru (M.C.); e.bystrovskaya@mknc.ru (E.P.); g.pronina@mknc.ru (G.P.); e.chernikova@mknc.ru (E.C.); d.bordin@mknc.ru (D.B.); 2 Department of Faculty Therapy, Smolensk State Medical University of the Ministry of Health of Russia, 214019 Smolensk, Russia; n.dekhnich@mail.ru; 3 Department of Propaedeutics of Internal Diseases and Gastroenterology of the Faculty of Medicine, Russian University of Medicine, 127473 Moscow, Russia; 4 Department of Family Medicine and General Medical Practice, Tver State Medical University, 170100 Tver, Russia

**Keywords:** *Helicobacter pylori*, antibiotic resistance, methods for determining *H. pylori* resistance, molecular genetic diagnostics, phenotypic methods for determining antibiotic resistance, geographical distribution of *H. pylori* antibiotic resistance

## Abstract

The Maastricht VI/Florence consensus recommends, as one of the measures to enhance the efficacy of *Helicobacter pylori* infection eradication, a personalized treatment approach involving the selection of an antimicrobial agent based on the pre-determined resistance of *H. pylori.* To address the need to develop test systems for personalized drug selection, this study was designed to analyze the molecular resistance of *H. pylori* using a newly developed Sanger sequencing test platform. The characteristics of the test system were determined on 25 pure culture samples of *H. pylori* with known resistance. Sensitivity and specificity for detecting resistance to clarithromycin was 100% and those to levofloxacin were 93% and 92%, respectively. The test system has been tested in real clinical practice on 112 *H. pylori*-positive patients who had not previously received proton pump inhibitors (PPIs) or antibacterial drugs. Mutations indicating resistance to clarithromycin were found in 27 (24%) samples and those indicating resistance to levofloxacin were found in 26 (23%) samples. Double resistance was observed in 16 (14%) samples. The most common mutations leading to clarithromycin resistance were 2143G and 2142G and to levofloxacin resistance—261A and 271A in the *gyrA* gene, which account for 69% of all identified genetic determinants in levofloxacin-resistant bacteria. Thus, a personalized approach to the selection of *H. pylori* eradication therapy based on the detection of bacterial resistance before prescribing first-line therapy could help to avoid the prescription of ineffective *H. pylori* eradication therapies and, overall, contribute to the control of antibiotic resistance of *H. pylori*.

## 1. Introduction

The issues of diagnosis and treatment of *Helicobacter pylori* infection have attracted the attention of the scientific community worldwide, primarily due to its high prevalence [1,2,3]. *H. pylori* infection in all cases leads to chronic gastritis and is the main etiological factor involved in gastric adenocarcinoma, including proximal gastric cancer [4,5,6,7].

Detecting and eradicating *H. pylori* is a key factor in the primary prevention of gastric cancer [8]. The efficacy of eradication regimens has decreased due to the rising resistance of *H. pylori* to antibiotics, particularly clarithromycin and levofloxacin [9].

According to the European Registry on *Helicobacter pylori* Management (Hp-EuReg), the effectiveness of classic triple therapy in Russia is only 80%, with a prescription rate of 56% [10]. This is largely attributed to the high level of *H. pylori*’s resistance to clarithromycin. According to the Hp-EuReg, the resistance of *H. pylori* to clarithromycin in Russia is 24%, its resistance to levofloxacin—27%, and its resistance to metronidazole—29% [11]. A recently published meta-analysis demonstrated a decrease in the global prevalence of *H. pylori* from 58% to 43% between 1980 and 2022 [12]. In Russia, the prevalence of *H. pylori* according to the ^13^C urea breath test was around 40% from 2017 to 2019 [13].

The increase in *H. pylori* resistance to the main antibiotics used in eradication regimens has led to changes in the principles of the selection of therapy. The goal of eradication therapy is to reliably cure *H. pylori* infection in the majority (≥90%) of patients, which requires the use of antibacterial agents to which the infection is susceptible, considering regional (local) data. The strategy for selecting eradication therapy based on local data was developed in 2022 in a consensus report [14]. Physicians obtain information about *H. pylori’s* resistance to antimicrobial agents through several methods. The first method involves culturing with subsequent sensitivity testing to antibiotics or molecular testing. The second method involves assessing the prevalence of resistance in other microorganisms in the community, such as respiratory pathogens to clarithromycin. The third method, accessible to all, involves conducting and organizing data from control tests after eradication and selecting the most effective empirical therapy regimens in the given population. Failure of treatment with an optimized modern regimen may clearly indicate the presence of resistance and that such therapy should no longer be recommended or used if local sensitivity is not confirmed by culture or molecular testing.

Perhaps the presence of well-known antibacterial agents that are familiar to practicing specialists should be considered the most important. Rational antibacterial therapy and a focus on determining the current sensitivity of *H. pylori* to antimicrobial agents are the priorities for effective eradication therapy. This allows, firstly, to avoid prescribing ineffective antimicrobial drugs. Secondly, it contributes to restraining the growth of *H. pylori* antibiotic resistance. Thirdly, it reduces the overall burden of antimicrobial drugs on the population [15,16].

The Taipan Consensus (2020) suggests considering the profile of *H. pylori* antibiotic resistance, the efficacy of antibiotics, side effects, and therapy cost when choosing optimal treatment regimens for the population [17]. Due to the increasing antibiotic resistance of *H. pylori*, the Maastricht VI/Florence consensus recommends conducting sensitivity tests (molecular or bacteriological) in a planned manner, even before prescribing first-line therapy, to ensure rational antibiotic use [14].

According to literature data, clarithromycin resistance is mainly caused by three-point mutations in the *23S rRNA* gene: A2142G, A2142C, and A2143G, encoding peptidyl transferase in the V domain of the gene, which is the main target of macrolides. Point mutations in this region disrupt the binding of macrolides to bacterial cell ribosomes, leading to resistance formation [18,19,20,21,22,23,24]. *23S rRNA* is found in the 50S subunit of prokaryotic ribosomes and is characterized by gene redundancy. The gene copy number of *23S rRNA* can vary from 1 to 15 in different prokaryotes. *H. pylori* has two copies of the *23S rRNA* gene, and if a mutation is present in at least one of these copies, the bacterium is considered resistant to clarithromycin [25].

Resistance to fluoroquinolones is caused by mutations in the *gyrA* gene, which encodes the A subunit of bacterial DNA gyrase, mainly at codons 87, 88, and 91 of the *gyrA* gene [18,19,20,21,22,23,24]. In different regions, the frequency and nature of mutations that confer *H. pylori* resistance can differ [26].

All existing recommendations today emphasize the preference for the personalized selection of antibiotic therapy based on the pre-treatment determination of *H. pylori’s* antibiotic resistance. This determination can be based on a phenotypic method that has high accuracy but is labor intensive, expensive, and requires specialized technical equipment. To assess resistance, it is necessary to first culture *H. pylori* from a gastric mucosa biopsy and then determine antibiotic sensitivity. This approach is difficult to implement in routine diagnostic laboratory practices. From an economic perspective, a PCR test for *H. pylori* antibiotic sensitivity is promising. However, the development of PCR diagnostic test systems for *H. pylori* antibiotic resistance should be based on population data about the regional characteristics of bacterial molecular resistance. Analyzing the genetic determinants responsible for clarithromycin and levofloxacin resistance in *H. pylori* in the Russian Federation is a necessary step for creating a valid diagnostic test system.

The purpose of this study is to study the molecular resistance to clarithromycin and levofloxacin in Moscow, Russia.

## 2. Materials and Methods

### 2.1. Test System Development

Based on the analysis of literature data regarding the genetic determinants of *H. pylori* antibiotic resistance, a Sanger sequencing test system for regions of the genes 23S rRNA and *gyrA*, containing the resistance targets to clarithromycin and levofloxacin, respectively, was developed at the A.S. Loginov Moscow Clinical Scientific Center. The study consisted of two stages. In the first stage of the study, the developed test system was tested using 25 samples of pure cultures with known phenotypic resistance to clarithromycin and levofloxacin. The results were compared, and the sensitivity and specificity of the method were determined [27]. In the next stage, biopsy samples from *H. pylori*-positive patients were sequenced to search for mutations in the *23S rRNA* and *gyrA* genes.

### 2.2. Strain Phenotypic Testing

Phenotypic determination of 25 *H. pylori* strain antibiotic resistance was carried out at the Smolensk State Medical University’s Research Institute of Antimicrobial Chemotherapy using the serial dilution method in Mueller–Hinton cation-adjusted agar (OXOID, Basingstoke, UK) with the addition of sheep blood (final concentration 5%) (E&O Laboratories Ltd., Burnhouse, UK) following the recommendations of the Clinical and Laboratory Standards Institute (CLSI) [28]. Criteria for assessing *H. pylori* sensitivity to antimicrobial drugs, as presented in Table 1, were used.

Subsequent molecular genetic determination of the resistance 25 *H. pylori* strain was performed at the A.S. Loginov Moscow Clinical Scientific Center using the Sanger sequencing method. The study was conducted blindly: after allele variants in antibiotic resistance genes were identified, the data on phenotypic resistance were revealed, and a comparative analysis of molecular genetic data with phenotypic data was conducted. The sequences were deposited in the NCBI Genbank https://www.ncbi.nlm.nih.gov/genbank/ (accessed on 7 June 2024). The sequences were assigned inventory numbers, which are included in the Supplementary Materials.

### 2.3. Test System Performance Evaluation

To identify the genetic characteristics of *H. pylori* antibiotic resistance, samples of gastric mucosa were analyzed, obtained during esophagogastroduodenoscopy (EGD) in 112 *H. pylori*-positive individuals in 2022–2023 as part of the “Epidemiological study of *H. pylori* infection prevalence in Moscow” grant funded by the Moscow Center for Innovative Technologies in Healthcare of the Moscow Healthcare Department, grant No. 0903-1/22, 21 March 2022. Before EGD, the patients underwent a C^13^-urea breath test.

The ages of the participants ranged from 18 to 80 years. All patients provided informed consent for the processing of personal data. The study was approved by the local ethics committee (approved by the Ethics Committee in A.S. Loginov Moscow Clinical Scientific Center, protocol No. 4/2022, 21 April 2022).

DNA extraction was performed using the commercial “Probe NK” kit (DNA-Technology, Moscow, Russia). Amplification was carried out using specific primers for the regions of the *23S rRNA* and *gyrA* genes on a T100 thermocycler (Bio-Rad, Hercules, CA, USA) in a reaction mixture of 50 µL, including: 67 mM Tris-HCl (pH = 8.8), 1.5–2.5 mM MgCl_2_, 4 ng genomic DNA, 5 pM of each primer, 10 mM of dATP, dGTP, dCTP, and dTTP, and 5 units of active Taq polymerase, following this scheme: (1) denaturation at 95 °C for 5 min; (2) denaturation at 95 °C for 30 s; primer annealing at 60–66 °C for 30 s; elongation at 72 °C for 15 s; and (3) final elongation at 72 °C for 7 min. The primer sequences developed for this study are shown in Table 2. Qualitative analysis of PCR product accumulation was performed using electrophoresis on a 2% agarose gel. Subsequent purification of the PCR product was performed using columns (Eurogene, Moscow, Russia). Sequencing reactions were performed with forward and reverse primers using the BigDye Terminator v3.1 Cycle Sequencing Kit (Thermo Fisher Scientific, San Diego, CA, USA) according to the manufacturer’s protocol. The sequencing products were then purified using the BigDye Terminator Kit (Thermo Fisher Scientific, San Diego, CA, USA). Detection of sequencing products was performed using capillary gel electrophoresis on an 8-capillary genetic analyzer 3500 (Genetic Analyzer 8ch, Thermo Fisher Scientific, Waltham, MA, USA). Electropherograms were analyzed using Chromas software (version 2.0.0.0, Technelysium, Australia) and Sequencing Analysis Software 7 (Thermo Fisher Scientific, USA). The identity of the amplified fragments was confirmed in the BLAST NCBI system. Sequences with a length of 255 base pairs were tested in the NCBI BLAST system with a 97% match percentage with the *Helicobacter pylori* genome.

## 3. Results

### 3.1. Test System Development

In the first stage of the study, sequencing data were obtained for regions of genes associated with resistance to clarithromycin and levofloxacin—*23S rRNA* (2142A > G, 2142A > C, and 2143A > G) and *gyrA* (codons 87 and 91), respectively, from twenty-five pure cultures of *H. pylori*. It was found that 11 (44%) out of the 25 samples had mutations in the clarithromycin resistance gene, and mutations in the levofloxacin resistance gene were detected in 13 (52%) samples, with 6 (24%) samples exhibiting dual resistance. Genetic variants in the *23S rRNA* and *gyrA* resistance genes for the 25 *H. pylori* culture samples are presented in Table 3.

**Figure 1:**
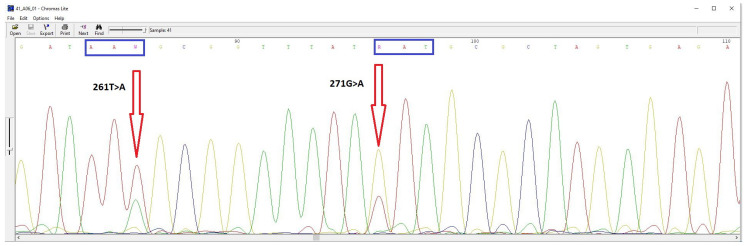
DNA sequencing chromatogram of the gyrA gene fragment from *H. pylori* cultures demonstrating genetic heterogeneity at position 271G/A.

It is worth noting that in some cultures, both wild-type and mutant alleles were detected (Figure 1). Most commonly, both alleles were found in the *gyrA* gene, which is responsible for levofloxacin resistance. The presence of two allelic states may be due to the genetic heterogeneity of *H. pylori*.

Comparative analysis of molecular and phenotypic resistance data for *H. pylori* showed complete concurrence between molecular–genetic and phenotypic resistance to clarithromycin. All 11 cultures with phenotypic resistance to clarithromycin had mutations in the *23S rRNA* gene. The analysis of levofloxacin resistance showed that 13 out of 14 cultures with phenotypic resistance to levofloxacin had mutations in the *gyrA* gene (Table 4). It is possible that in addition to mutations in codons 87 and 91, levofloxacin resistance may be caused by other *gyrA* gene mutations or mutations in other genes, requiring further research on levofloxacin resistance.

The sensitivity and specificity of the Sanger sequencing method for determining clarithromycin resistance were 100%, while for levofloxacin resistance, they were 93% and 92%, respectively. These findings indicate the high diagnostic value of the Sanger sequencing method, allowing for the determination of *H. pylori*’s resistance to clarithromycin and levofloxacin.

### 3.2. Testing of Patient Samples

Sequencing results using the Sanger method for 112 biopsies from *H. pylori*-positive patients allowed us to establish the spectrum of mutations reflecting resistance to clarithromycin and levofloxacin. Mutations in the 23S rRNA clarithromycin resistance gene were detected in 27 (24%) samples and mutations in the levofloxacin resistance gene gyrA in were detected 26 (23%) samples. At the same time, double resistance was detected in 16 (14%) samples; 59 (52%) of the studied samples did not have the corresponding mutations in the resistance genes to clarithromycin and levofloxacin.

#### 3.2.1. Molecular Resistance to Clarithromycin

The frequency and spectrum of identified mutations in the *23S rRNA* gene in this study are presented in Table 5. The most prevalent nucleotide substitution is 2143G at 18%, followed by the 2142G mutation at 5%, and the 2142C nucleotide variant at 0.9%. The investigation of the nature and spectrum of allele variants of the *23S rRNA* gene in Russia should continue. However, it is already evident that the most characteristic mutation is the nucleotide substitution A2143G.

#### 3.2.2. Molecular Resistance to Levofloxacin

The most common nucleotide variants of the *gyrA* gene were 261 A in codon 87 and 271 A in codon 91. The frequencies and spectrum of *gyrA* gene mutations characteristic of Moscow are shown in Table 5. Molecular resistance to levofloxacin is represented by mutations in codons 87 and 91 equally.

## 4. Discussion

*H. pylori* always causes gastritis and is one of the main etiopathogenetic factors in the development of peptic ulcer disease—the primary etiological factor for gastric adenocarcinoma. Moreover, *H. pylori* is the main risk factor for mucosa-associated lymphoid tissue lymphoma.

Eradication therapy of *H. pylori* infection eliminates the active inflammatory response in chronic active non-atrophic gastritis, leads to a reduced risk of erosive-ulcerative lesions in the gastric and duodenal mucosa, serves as a preventive measure against the development and progression of precancerous changes in the gastric mucosa (atrophic gastritis, intestinal metaplasia), and therefore acts as a primary preventer of stomach cancer. The main reason for the decreasing effectiveness of eradication therapy regimens is the formation and increasing resistance of *H. pylori* to antibiotics. This phenomenon is a consequence of the use of ineffective eradication therapy regimens and the widespread use of macrolides and fluoroquinolones for various indications, leading to the development of corresponding mutations in *H. pylori* genes. The latest international consensus, Maastricht VI/Florence, recommends an individualized prescription of eradication regimens, taking antibiotic resistance into account, as well as empirical therapy considering regional variations in resistance and therapeutic efficacy. These recommendations allow for the implementation and increased accessibility of methods for determining *H. pylori* antibiotic resistance, both phenotypic and molecular–genetic.

When it comes to the effectiveness of *H. pylori* eradication, it is not solely determined by the sensitivity of the bacteria to antibiotics; this is just one of the factors influencing the effectiveness of anti-*Helicobacter* therapy. Overall, the effectiveness of anti-*Helicobacter* therapy depends on factors related to the bacterium itself, such as antibiotic resistance, strain virulence, bacterial load, and the ability to form biofilms. It also depends on patient-related factors, including compliance, hypersecretion of gastric acid, and polymorphisms in genes responsible for the metabolism of various drugs used in *H. pylori* eradication. An important aspect of *H. pylori* infection and the treatment of other gastric diseases is the treatment strategy. A well-thought-out, comprehensive, and personalized approach tailored to individual patient characteristics will contribute to treatment efficacy. Currently, the pharmacogenetic features of patients are actively being studied, which can improve treatment outcomes by allowing a targeted approach to therapy selection and reducing side effects. Exploring a patient’s pharmacogenetic profile can help avoid significant costs associated with expensive drugs when they are ineffective and require prolonged treatment.

International guidelines highlight the relevance of developing and implementing molecular diagnostic methods for *H. pylori* infection and assessing its susceptibility to antibiotics, primarily clarithromycin and levofloxacin. Molecular methods, such as real-time PCR, next-generation sequencing (NGS), and digital PCR, allow for the detection of mutations in genes associated with resistance to these antibiotics. Additionally, the development of technologies for detecting genes associated with resistance to metronidazole, tetracycline, and rifaximin is a promising area of research.

For molecular testing, e.g., using PCR, biopsies of the gastric mucosa are suggested to be used, including those extracted from urease tests (rapid urease test). This technology enables the assessment of antibiotic resistance in previously untreated patients and after unsuccessful eradication, forming the basis for individualized therapy selection. Moreover, the availability of resistance detection technologies allows for evaluating the prevalence of resistant strains in the region. This information is crucial for forming recommendations for knowledge-based therapy selection.

The molecular–genetic method for determining *H. pylori’s* antibiotic resistance demonstrates high sensitivity and specificity. The sensitivity and specificity of the molecular–genetic method for determining clarithromycin resistance were 100%, and for levofloxacin resistance, they were 93% and 92%, respectively. According to the literature, the sensitivity and specificity of the molecular–genetic method for clarithromycin resistance determination are reported to be 93% and 98%, and for levofloxacin resistance, they are 83% and 94%, respectively [29]. However, 25 cultures of *H. pylori* are not sufficient to convincingly confirm the sensitivity and specificity of the method used. It is necessary to continue the study on a larger number of samples with known phenotypic resistance to antibiotics.

In biopsies obtained during EGD, in the period of 2022–2023, molecular resistance to clarithromycin was detected in 24% of cases, resistance to levofloxacin was detected in 23% of cases, including double resistance in 14% of cases, and 59 (52%) of the studied samples did not have the corresponding mutations in the resistance genes to clarithromycin and levofloxacin.

It should be noted that the Sanger sequencing method has limitations, and its sensitivity is around 20%. Therefore, if the test sample contains a mixture of *H. pylori* strains with a proportion of resistant bacteria less than 20%, there is a probability of a false negative result obtained using the Sanger sequencing method. In this case, the RT-PCR method becomes preferred.

The nature of substitutions that lead to *H. pylori*’s resistance to clarithromycin, levofloxacin, and other antibiotics varies across global populations. In this study, the most common mutations reflecting clarithromycin resistance were variants 2143G and 2142G, which is consistent with world data. However, in some world regions, different nucleotide variants are encountered. For example, variants 2142G and 2143G are characteristic of Iran, Tunisia, Vietnam, and Russia [21,30,31]. T248C is characteristic of Myanmar, while for Sudan, in addition to 2142G and 2143G, variants T2182C and C2195T are also found [32,33].

The most frequent mutations responsible for *H. pylori* resistance to levofloxacin among Moscow residents were variants 261A and 271A, which accounted for 69% of all identified genetic determinants of levofloxacin resistance in this study. It is worth noting that the spectrum and distribution pattern of allele variants of the *gyrA* gene slightly differ from global data, where the most common mutations at codon 87 of the gyrA gene are reported to be 259T and 261A. For instance, the 259T mutation of the gyrA gene in *H. pylori* among Moscow residents has been very rarely observed (0.9%), whereas the variant 260T was encountered at the same frequency (0.9%).

For example, in the population of Myanmar, almost all levofloxacin-resistant isolates had an amino acid substitution at position 91 (Asp-91 to Asn or Tyr) and had no substitutions at codon 87. However, levofloxacin-resistant strains in neighboring countries of Southeast Asia, such as Indonesia, Malaysia, and Cambodia, had both mutations [33]. Many studies have described single or double mutations at positions Asn87 and Asp91 as the most common mutation sites in resistant *H. pylori* isolates obtained from the gastric mucosa [34,35,36].

In the United States, common gene *23S rRNA* substitutions include variants 2142 and 2143, while typical mutations of *gyrA* are 260T, 261A, 271A, and 271T [29]. In northern Israel, *23S rRNA* gene variants 2142G, C2173T, and G2212A were exclusively detected in phenotypically resistant *H. pylori* isolates. At the same time, variant 2143G was found in both resistant and susceptible *H. pylori* isolates. As for the levofloxacin resistance gene, mutations at codons 87 and 91 were found in *H. pylori* isolates in northern Israel at a frequency of 4.2% for both resistant and susceptible isolates, while the *gyrA* T239M variant was detected in phenotypically resistant isolates [37]. The results of conducted studies demonstrate genetic specificity regarding the molecular resistance of *H. pylori* to antibiotics.

An important aspect that must be considered in the molecular diagnosis of *H. pylori* antibiotic resistance is the genetic heterogeneity of bacteria inhabiting the gastric mucosa, when different strains of *H. pylori*, both sensitive and resistant, coexist in the patient’s gastric mucosa. In this regard, it is necessary to examine several fragments of the gastric mucosa from one patient, which will increase the accuracy of the diagnosis of antibiotic resistance.

## 5. Conclusions

A high detection level of *H. pylori* resistance to clarithromycin and levofloxacin in Moscow was demonstrated, comparable to recent European studies. Additionally, specific features of the spectrum and the nature of genetic determinants of molecular resistance of *H. pylori* to these antibiotics were identified.

According to current recommendations, eradication of *H. pylori* in adults is indicated in all cases, primarily for the primary prevention of gastric cancer. One of the strategies that enhances the effectiveness of therapy in the context of high antibiotic resistance is personalized antibiotic selection based on determining *H. pylori* resistance before administration. Investigating *H. pylori*’s resistance to antibiotics also provides a rationale for choosing knowledge-based therapy based on monitoring local *H. pylori* resistance to clarithromycin.

## 6. Patents

Patent No. 2806581 dated 1 November 2023.

## Figures and Tables

**Table 1:** Criteria for assessing the sensitivity of *H. pylori* to antimicrobial drugs using the phenotypic method.

Antibiotic	Minimum Suppressive Concentration, mg/L	
	Sensitive ^1^, ≤	Resistant ^1^, >
**Clarithromycin**	0.25	0.5
**Levofloxacin**	1.0	1.0

*Note:* ^1^ These criteria are not clinical but represent epidemiological threshold values that distinguish strains with natural sensitivity from strains with reduced sensitivity.

**Table 2:** Sequences of primers used.

Gene	The Primer Sequence	Length (bp)
23S rRNA	F: 5′-TAAATACCGACCTGCATGAATG-3′	22
	R: 5′-CTCCATAAGAGCCAAAGCCC-3′	20
gyrA	F: 5′-ATCATAGGGCGTGCTTTACC-3′	20
	R: 5′-AAAGGTTAGGCAGACGGCTT-3′	20

**Table 3:** Frequency of occurrence of genetic variants of *H. pylori* antibiotic resistance genes (rRNA *S23*, *gyrA*) in 25 *Helicobacter pylori* cultures.

Antibiotic Resistance	Gene	Nucleotide Substitution	N (%)
Clarithromycin	*23S rRNA*	2142G	7 (28)
		2143G	4 (16)
		WT *	14 (56)
Levofloxacin	*gyrA*	261A	3 (12)
		261G	1 (4)
		271A	4 (16)
		272G	3 (12)
		261A, 271A	2 (8)
		WT *	12 (48)
Both	*23S, gyrA*	–	6 (24)

*Note:* * WT—wild type.

**Table 4:** Comparison of *H. pylori* resistance according to phenotypic and molecular methods.

Antibiotic	Genetic Testing		Phenotypic Testing	
	Resistant	Sensitive	Resistant	Sensitive
Clarithromycin	11	14	11	14
Levofloxacin	13	12	14	11
Total	25		25	

**Table 5:** Frequency and spectrum of mutations in the 23S rRNA and *gyrA* genes in a study of 112 *Helicobacter*-positive patients in Moscow (%).

Antibiotic Resistance	Gene	Nucleotide Substitution	N (%)
Clarithromycin	*23S rRNA*	2142G	6 (5)
		2143G	20 (18)
		2142C	1 (0.9)
		WT *	85 (76)
Levofloxacin	*gyrA*	259T	1 (0.9)
		260T	1 (0.9)
		261A	11 (9.8)
		271A	7 (6.3)
		271T	4 (3.5)
		272G	2 (1.8)
		WT *	86 (76.8)

*Note:* * WT—wild type.

## Data Availability

The data are available from the corresponding author upon request.

## Supplementary Materials

The following supporting information can be downloaded at: https://www.mdpi.com/article/10.3390/cimb46070397/s1, sequence accession numbers in GeneBank.
