# Supplementary material for: Genetic Markers of Helicobacter pylori Resistance to Clarithromycin and Levofloxacin in Moscow, Russia
Source: Curr Issues Mol Biol. 2024 Jun 29;46(7):6665–74. doi: 10.3390/cimb46070397 (PMC11276122; doi:10.3390/cimb46070397)
Supplement: Supplementary file 1 [file cimb-46-00397-s001.zip › cimb-2983328-supplementary.pdf]

23S rRNA gene sequence accession numbers

SUB14452744 Seq1 PP803429
SUB14452744 Seq2 PP803430
SUB14452744 Seq3 PP803431
SUB14452744 Seq4 PP803432
SUB14452744 Seq5 PP803433
SUB14452744 Seq6 PP803434
SUB14452744 Seq7 PP803435
SUB14452744 Seq8 PP803436
SUB14452744 Seq9 PP803437
SUB14452744 Seq10 PP803438
SUB14452744 Seq11 PP803439
SUB14452744 Seq12 PP803440
SUB14452744 Seq13 PP803441
SUB14452744 Seq14 PP803442
SUB14452744 Seq15 PP803443
SUB14452744 Seq16 PP803444
SUB14452744 Seq17 PP803445
SUB14452744 Seq18 PP803446
SUB14452744 Seq19 PP803447
SUB14452744 Seq20 PP803448
SUB14452744 Seq21 PP803449
SUB14452744 Seq22 PP803450
SUB14452744 Seq23 PP803451
SUB14452744 Seq24 PP803452
SUB14452744 Seq25 PP803453

gyrA gene sequence accession numbers

BankIt2831128 Seq1 PP817812
BankIt2831128 Seq2 PP817813
BankIt2831128 Seq3 PP817814
BankIt2831128 Seq4 PP817815
BankIt2831128 Seq5 PP817816
BankIt2831128 Seq6 PP817817
BankIt2831128 Seq7 PP817818
BankIt2831128 Seq8 PP817819
BankIt2831128 Seq9 PP817820
BankIt2831128 Seq10 PP817821
BankIt2831128 Seq11 PP817822
BankIt2831128 Seq12 PP817823
BankIt2831128 Seq13 PP817824
BankIt2831128 Seq14 PP817825
BankIt2831128 Seq15 PP817826
BankIt2831128 Seq16 PP817827
BankIt2831128 Seq17 PP817828
BankIt2831128 Seq18 PP817829
BankIt2831128 Seq19 PP817830

BankIt2831128 Seq20 PP817831
BankIt2831128 Seq21 PP817832
BankIt2831128 Seq22 PP817833
BankIt2831128 Seq23 PP817834
BankIt2831128 Seq24 PP817835
BankIt2831128 Seq25 PP817836
